# Multimodal Sentiment and Emotion Analysis Framework for Personalized Health Coaching Messages: Proof-of-Concept Study

**DOI:** 10.2196/79558

**Published:** 2026-04-21

**Authors:** Muhammad Aiman Md Zuki, Nazlena Mohamad Ali, Jun Kit Chaw

**Affiliations:** 1 Institute of Visual Informatics (IVI), Universiti Kebangsaan Malaysia, Jalan Tun Ismail Ali, Bandar Baru Bangi, Selangor, 43600, Malaysia, 60 0389272402

**Keywords:** sentiment analysis, emotion detection, persuasive communication, health coaching, natural language processing

## Abstract

**Background:**

Text generation approaches in health care communication have evolved along 2 major paths. The first path involves generative adversarial networks, progressing from basic architectures to specialized variants like Text-to-Text Generative Adversarial Network (TT-GAN) and Time and Frequency Domain-Based Generative Adversarial Network (TF-GAN), which address challenges in discrete text generation through techniques such as Gumbel-Softmax and reinforcement learning. The second path emerges from transformer-based architectures, particularly Generative Pretrained Transformer-2 (GPT-2), which uses extensive pretraining and self-attention mechanisms to generate contextually appropriate text. GPT-2’s transformer architecture enhances persuasive health communication by generating personalized messages using various strategies like task support, dialogue support, and social support for effective health interventions.

**Objective:**

This study aimed to use GPT-2 as a generative method to construct persuasive text in a dataset and compare the performance of sentiment analysis and emotion detection analysis.

**Methods:**

We combined sentiment analysis tools (VADER [Valence Aware Dictionary and Sentiment Reasoner] and TextBlob) with emotion detection methods (Text2Emotion and NRCLex [National Research Council Lexicon]) to analyze health coaching messages across different persuasive types: reminder, reward, suggestion, and praise.

**Results:**

TextBlob and VADER achieved accuracies of 57% and 69%, respectively, while RoBERTa (robustly optimized BERT approach)-sentiment outperformed them with an accuracy of 88%. Emotion detection showed a high prevalence of “joy” and “happy” labels (93.69% positive skew). While transformers excel in accuracy, lexicon-based models like VADER offer a better performance-efficiency balance for real-time health communication systems. For emotion detection, all categories showed perfect accuracy (1.0), while trust showed mixed results, with precision, recall, and *F*_1_-score values ranging from 0.81 to 0.96. The emotion detection analysis revealed varying success rates across different emotions, with some categories, such as anger and neutral, showing reasonable performance and others, such as trust, showing mixed performance.

**Conclusions:**

This research contributes to understanding the emotional dynamics of persuasive health communication and highlights both the capabilities and limitations of current natural language processing tools in analyzing health-related persuasive messaging. This proof-of-concept study using synthetically generated data establishes a methodological framework for multimodal sentiment and emotion analysis. The findings require validation with real-world health coaching messages before clinical deployment.

## Introduction

### Background

Health coaching has been actively researched, and the results are expected to increase human life expectancy. However, with the advancement of technology and social media, the pattern of life is also changing. It appears that health care professionals are not directly involved, and the content does not always align with the user’s behavior or situation [1]. The necessity for advanced data preprocessing and analysis frameworks to improve system efficacy has been brought to light by the explosive growth of persuasive technology. The optimization of these components mostly depends on precise data processing and user behavior interpretation, even if contemporary persuasive systems use a variety of tactics (eg, reminders, rewards, suggestions, and praise). This study tackles the difficulties in converting raw data into useful insights by offering a thorough paradigm for preprocessing and customizing data in persuasive system design (PSD). Adherence to digital behavior change interventions is problematic, and some reasons for this include the absence of a social incentive from a health care practitioner and the presence of content that may not always be relevant to the user’s circumstances [2].

Advanced data preprocessing and analysis frameworks are vital for improving system effectiveness, as evidenced by the quick development of persuasive technology. As pointed out in a previous study [1], the absence of social incentives and personalization makes it difficult to adhere to digital behavior modification treatments. Although computerized interventions offer practical ways to provide behavioral assistance at a reasonable cost [3], their efficacy mostly depends on precise data processing and user behavior interpretation.

Changing habits and lifestyle choices can significantly improve health outcomes and help prevent early mortality; thus, it is important to design compelling messaging strategies for a digital health assistant that can help individuals stay committed to their prescribed health programs and behavioral changes [4]. However, persuasive communication, which utilizes reinforcement learning (RL), has been implemented only in the reminder portion of PSD, and thus, it does not fully utilize the PSD capabilities proposed previously [5]. Moreover, this could lead to the dataset being used only for the reminder portion. On the other hand, there is no segment for implementing praise, reward, etc. This limitation motivated us to conduct our experiments with data preprocessing using Text-to-Text Generative Adversarial Network (TT-GAN) [6] and text generation with knowledge transfer from Generative Pretrained Transformer-2 (GPT-2) [7]. Generative adversarial networks (GANs), which represent a class of artificial intelligence (AI) models capable of producing incredibly realistic synthetic data, have emerged as a significant innovation and demonstrated impressive progress in machine learning studies.

Our framework encompasses multiple data processing layers, beginning with fundamental preprocessing techniques for cleaning and standardizing raw user interaction data. This includes handling missing values, normalizing temporal data, and standardizing input formats across various data sources. The behavior analysis component uses pattern recognition algorithms to identify user engagement trends, activity cycles, and response patterns to different persuasive elements. These patterns are crucial for developing effective personalization strategies.

The classification system utilizes machine learning algorithms to categorize user behaviors and preferences, enabling dynamic adaptation of persuasive strategies. Natural language processing (NLP) techniques are integrated to analyze user feedback and responses, extracting sentiment and context to refine the system’s understanding of user needs. This multifaceted approach ensures that the persuasive elements are personalized, contextually appropriate, and timely.

Our research combines these components into a cohesive framework to address the gap between raw data collection and effective persuasive strategy implementation. Compared with traditional static approaches, the proposed system significantly improves user engagement and behavior change outcomes while maintaining scalability and real-time processing capabilities.

To close the gap between health care professionals’ involvement and the GPT-2 architecture, we conducted an experiment combining the GPT-2 method and data preprocessing to obtain a better result for the PSD element. In this experiment, we advanced text generation using GPT-2. Then, we compared the result with the sentiment analysis finding and performed validation with health care practitioners to increase its relevance.

The contributions of this experiment are as follows:

- Introduce the framework of RL and persuasive technology (PT) as the combination of human-computer interaction elements and machine learning
- Demonstrate the effectiveness of using GPT-2 as a generative method to construct persuasive texts in datasets for health coaching messages
- Compare the performance of multiple sentiment analysis tools (VADER [Valence Aware Dictionary and Sentiment Reasoner] and TextBlob) when applied to health coaching messages across different persuasive types (reminder, reward, suggestion, and praise)
- Evaluate and compare the effectiveness of different emotion detection methods (Text2Emotion and NRCLex [National Research Council Lexicon]) for analyzing emotional content in health-related persuasive communication.

This study serves as a proof-of-concept for integrating generative AI with sentiment and emotion analysis in health-coaching contexts. We acknowledge that our use of GPT-2–generated synthetic data provides controlled conditions for methodological development but has inherent limitations in ecological validity. The findings represent baseline performance under ideal conditions and should not be extrapolated directly to real-world deployment without further validation using authentic patient-provider communication. This study provides a foundational computational framework for analyzing the sentiment and emotional characteristics of persuasive health coaching messages. We focused on establishing the baseline performance of NLP tools and identifying emotional patterns across persuasive types. However, we did not evaluate whether these sentiment/emotion labels translate to improved message quality, safety, or user outcomes. Such validation requires clinical trials with patient participants, which represents future work beyond the scope of this computational methodology study. Our contribution is the establishment of the analytical infrastructure necessary for future outcome-based validation.

In the next section, we discuss related work. Later, we provide some background on the knowledge used in the experiment and analyze the results. Lastly, we summarize the findings, provide conclusions, and indicate future directions.

### Related Work

#### Overview

GANs are AI models in which 2 neural networks compete against each other—one generates fake data while the other tries to detect the fake data. Over several years of study and experimentation, many variations of GANs have been improved in terms of flexibility (f-GAN [8]), stable training behavior compared with a traditional GAN (w-GAN [9]), and learning of disentangled and interpretable representations (Information-Theoretic Generative Adversarial Network [InfoGAN] [10]). However, TT-GAN appears more appropriate than the stated GANs, as it is more reliable for language and transformer-based models when using teacher forcing. With regard to dialogue generation, document summarization, and machine translation, language models play crucial roles in optimizing linguistic structure. Based on a previous report [7], existing neural networks can be broadly categorized into 2 types: recurrent models (eg, recurrent neural network [RNN] and long short-term memory [LSTM]) and self-attention models (eg, GPT-2 and Transformer-XL).

#### Text Generation Using GANs

A previous study [6] demonstrated that TT-GAN could successfully provide 2 types of text outputs: semantic summarization and paraphrasing of movie reviews in both English and Chinese. However, an examination of the experiments reveals a lack of a precise comparative analysis between the proposed approach and existing methods in terms of limitations.

A previous survey [11] identified 3 main approaches to GAN-based text generation: Gumbel-Softmax differentiation, RL, and modified training objectives. The adaptation of GANs for text generation presents several challenges, including the initial design of GANs for continuous data (eg, images) and not discrete text data; the need to preserve grammar, syntax, and semantic properties; and the pretraining burden for many approaches. In terms of language structure requirements, it is important to preserve grammatical accuracy and maintain syntactic coherence. This can lead to the challenge of connecting sequences logically. Finally, technical limitations regarding memory consumption and context understanding remain persistent obstacles when adapting GANs for generating text.

Time and Frequency Domain-Based Generative Adversarial Network (TF-GAN), which has been proposed previously [7], transforms the active learning process from operating on discrete text elements to working within a continuous space of text features by utilizing maximum likelihood estimation (MLE) to convert features back into tokens. Because the MLE model is not an issue, TF-GAN can produce diverse, high-quality text outputs while maintaining variety in its generations.

InfoGAN is considered superior to other GAN methods for several key reasons. First, it uniquely achieves high-quality disentangled representations in a completely unsupervised manner, while previous approaches require supervision. Second, it successfully disentangles meaningful features like writing styles, pose, lighting, and facial attributes across various datasets (MNIST, 3D faces, SVHN, and CelebA) with quality that matches or exceeds supervised methods. Third, its information-theoretic approach of maximizing mutual information between latent codes and observations naturally discovers important data variations without supervision.

#### GPT-2 for Implementation

Using sophisticated computational methods, text generation systems can produce novel content in response to prompts, creating outputs that frequently appear similar to human writing. Language models that perform the best on linguistic tasks combine 2 key steps: initial pretraining and supervised fine-tuning [12]. Recent research indicates that specialized architectural designs for individual tasks may be unnecessary, and the use of multiple self-attention layers and their transfer are adequate [13,14].

In the context of health, when doctors are not available, health care workers can struggle to provide patients with complete information about their medications, including the benefits and side effects. The ability of GPT-2 to generate summaries could help address this information gap [15]. The study examined whether GPT-2 could accurately understand and explain medication instructions when provided with specific medication names as input [15]. A dataset from the PubMed National Library of Medicine was used for the assessment.

On comparing the proposed Generative Pretrained Transformer (GPT) model implementation for text generation in the health care domain with other methods (eg, InfoGAN and TT-GAN), the proposed approach appears to have some distinct advantages and tradeoffs. While InfoGAN focuses on learning disentangled representations in an unsupervised manner through mutual information maximization and TT-GAN is aimed at paraphrase generation using adversarial networks, the GPT-based method, specifically in [15], targets health care text generation with a more straightforward architecture.

The evolution from simple GANs to more sophisticated models like GPT demonstrates significant progress but also highlights the need to evaluate the generated content carefully. There are various GAN architectures for text generation, but their comparative performance metrics must be critically examined. While TT-GAN’s capabilities in semantic summarization and paraphrasing have been mentioned [15], the challenges in text generation reveal significant gaps between theoretical models and practical implementation. The transition from traditional GANs to TF-GAN shows the evolution in handling discrete text data, yet questions remain about the tradeoffs between model complexity and output quality. While relevant, the inclusion of GPT-2 applications in health care appears disconnected from the main discussion of GAN-based text generation, suggesting a need for a more cohesive analysis of different generative approaches. Despite being outdated, GPT-2 still has value for certain applications, such as data augmentation and exploration of text generation concepts. GPT-2 is capable of producing excellent, grammatically sound, and semantically cohesive text. It can produce long-form content that is frequently indistinguishable from human-written text, such as essays, articles, and stories. GPT-2 has shown competence across diverse tasks like summarization, question answering, and text completion [16]. In the experiments, we used GPT-2 as the model for working with persuasive elements, serving as a baseline for future improvements. This establishes a benchmark for comparison with newer models in terms of performance, constraints, and other aspects in health care settings.

Table 1 compares TF-GAN, InfoGAN, and GPT-2. Based on the findings, GPT-2 was chosen as the generating agent for the dataset. As noted by Karak et al [15], GPT models demonstrate superior performance in generating health care–appropriate content, particularly for patient communication tasks, which are similar to our health coaching scenario. While newer models exist, GPT-2 provides an established baseline for comparison and demonstrates the viability of transformer-based approaches over adversarial networks in this domain.

**Table 1.** Comparative analysis of GPT-2^a^ and GAN^b^-based models for health text generation.

Criterion	TF-GAN^c^	InfoGAN^d^	GPT-2
Architecture	Adversarial (generator-discriminator)	Adversarial with mutual information maximization	Transformer with self-attention
Training stability	Prone to mode collapse and instability	Requires careful balancing of objectives	Stable autoregressive training
Text coherence	Struggles with long-form semantic coherence	Limited to feature disentanglement	Maintains grammatical and semantic consistency
Controllability	Requires complex conditional architecture	Unsupervised learning of latent codes	Direct prompt-based control for persuasive types
Domain adaptation	High pretraining burden for health care	Not designed for text generation tasks	Fine-tuning on health communication
Output diversity	Achieved through continuous space transformation	Good for representation learning	Natural diversity through temperature sampling
Health care suitability	Complex discrete-to-continuous conversion	Better for image/feature analysis	Proven effectiveness in medical text generation
Implementation complexity	High (adversarial training dynamics)	High (information-theoretic optimization)	Low (straightforward fine-tuning)
Baseline value	Limited health care text applications	Not applicable to text generation	Established baseline for transformer comparisons

a GPT-2: Generative Pretrained Transformer-2.

b GAN: generative adversarial network.

c TF-GAN: Time and Frequency Domain-Based Generative Adversarial Network.

d InfoGAN: Information-Theoretic Generative Adversarial Network.

#### Emotion Detection Analysis

In previous years, researchers explored 2 main approaches for detecting emotions in text: one approach used machine learning algorithms to train emotion detection models, and the other approach relied on lexicons (collections of words that typically express specific emotions) [17]. The discrete emotion model organizes emotions by sorting them into separate and distinct categories or classes. Like the Ekman theory, the Plutchik model proposes that a limited number of basic emotions exist in pairs of opposites, and these primary emotions can combine to create more complex emotional states [18].

**Figure 1.**
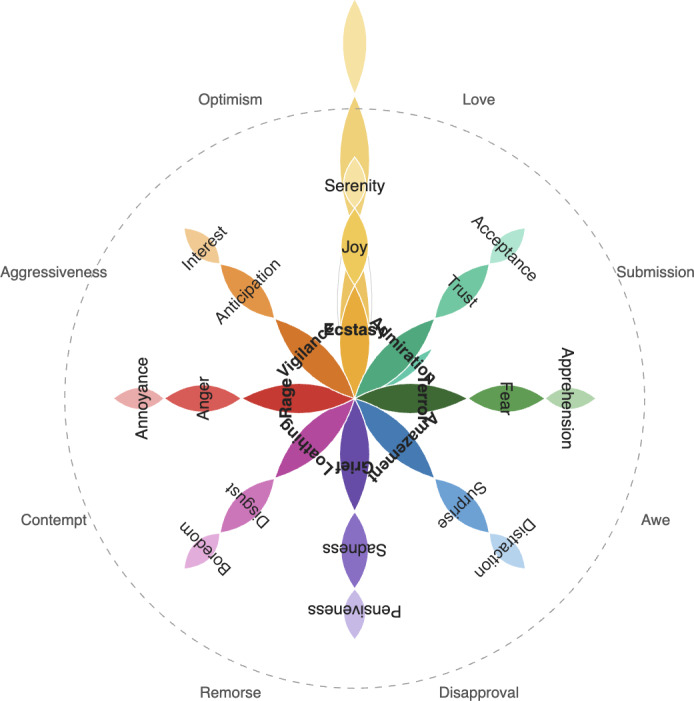
Plutchik emotion wheel [19].

The approach involves comprehensive emotion analysis of a given text message using 2 different emotion analysis libraries. The experiment implemented NRCLex emotion detection, which is the same as Plutchik emotion dyads (anticipation, joy, trust, fear, surprise, sadness, disgust, and anger; Figure 1) [19], but with the addition of positive and negative.

### PSD Framework

PSD provides the theoretical foundation for developing systems that influence user attitudes and behaviors through persuasion rather than coercion. Oinas-Kukkonen and Harjumaa [5] established the seminal PSD framework, identifying 28 design principles organized into 4 categories: primary task support, dialogue support, system credibility support, and social support. Among these, dialogue support principles are particularly relevant to health coaching applications, encompassing praise, reward, reminder, suggestion, similarity, liking, and social role elements.

The application of PSD to health behavior change has evolved significantly, yet critical gaps remain in existing implementations. Early health coaching systems focused primarily on single persuasive strategies. Beinema et al [1] implemented embodied conversational agents with automatic topic selection but primarily utilized reminder-based approaches. op den Akker et al [2] proposed the Council of Coaches framework for holistic behavior change, incorporating multiple PSD elements but facing implementation complexity challenges. Most significantly, Albers et al [4] developed an RL approach for persuasive conversational agents but implemented only the reminder component of dialogue support, leaving other PSD elements (praise, reward, and suggestion) unexplored in their dataset and system.

This limited implementation represents a significant gap in PSD research. The dialogue support category encompasses multiple complementary strategies, with each serving distinct psychological functions: reminders prompt action, praise provides positive reinforcement, rewards acknowledge achievements, and suggestions offer guidance. By focusing exclusively on reminders, previous research has not fully utilized the capabilities proposed by the PSD framework or examined how different dialogue support elements may carry varying emotional and sentiment characteristics. This research contributes to PSD literature by providing empirical evidence for how different dialogue support elements function emotionally and sentimentally. The finding that praise naturally carries higher emotional intensity than reminders or suggestions validates intuitive assumptions in PSD theory while providing quantifiable metrics for these differences. Understanding these emotional signatures enables more sophisticated PSD, where different PSD elements can be strategically deployed based on desired emotional impact and user state.

## Methods

### Study Design and Overview

This is a computational methodology development study (not a clinical trial) using synthetically generated health coaching messages (n=1300). No human participants or real patient data were involved. The primary objective was to validate sentiment and emotion analysis tools for health-specific persuasive communication.

**Figure 2.**
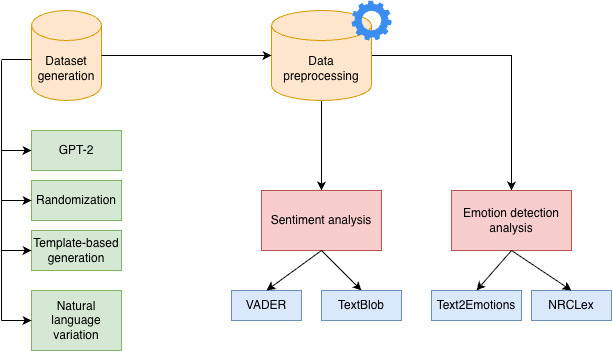
Process of the experiment. GPT-2: Generative Pretrained Transformer-2; NRCLex: National Research Council Lexicon; VADER: Valence Aware Dictionary and Sentiment Reasoner.

We constructed the dataset using GPT-2, randomization, natural language variation, and template-based generation (Figure 2). This section will explain the step-by-step process of this experiment. In the first stage, the dataset was constructed using GPT-2 and Python. The second stage involved data preprocessing, including tokenization, stemming, lemmatization, etc. Subsequently, we performed sentiment analysis using VADER and TextBlob. When compared to complex machine learning methods, VADER’s straightforward approach offers notable benefits. It provides fast and efficient processing while maintaining high accuracy levels. Additionally, unlike machine learning models, where the decision-making process is obscured in a black box, VADER’s dictionary and governing rules are transparent and can be directly examined [20]. As a result, VADER can be readily reviewed, comprehended, expanded, or customized according to specific needs. TextBlob, implemented as a Python library, provides an uncomplicated interface to execute fundamental NLP operations. One of its key advantages is that it handles text much like Python strings, making it particularly user-friendly and straightforward to implement [21].

### Data Generation

In the first phase of creating the dataset, we used GPT-2 for the pretrained dataset. We loaded the GPT-2 tokenizer and model using “tokenizer=GPT2Tokenizer.from_pretrained(‘gpt2’)” and “model=GPT2LMHeadModel.from_pretrained(‘gpt2’),” respectively. Activities for weight loss, such as water intake [22], portion control, meal planning, and healthy eating [23], were set to predefined activities. Then, we prompted text generation by categorizing it as reminder, reward, suggestion, and praise.

We used randomization, template-based generation, and natural language generation techniques to make it more interesting and natural. This dataset was artificially generated using GPT-2. However, the dataset is based on previous research [4]. It has been indicated that the dataset only used reminder as its PT element. Thus, we created a new dataset containing more PT elements (reminder, reward, suggestion, and praise). Based on a previous report [24], the use of at least 1000 samples in the dataset generally produces acceptable and reliable performance results when testing NLP models.

Table 2 provides examples demonstrating that GPT-2 successfully generated diverse messages across persuasive types while maintaining semantic coherence and health-appropriate content, validating our choice of generative approach.

**Table 2.** Examples demonstrating GPT-2^a^ diversity and generalization.

Persuasive type and activity		Example message	Diversity feature
Reminder	
Water intake		Don’t forget to drink your water today! Staying hydrated helps your body function at its best.	Standard instructional tone
Meal planning		Time to plan your meals for the week. A little preparation goes a long way!	Task-focused approach
Reward	
Portion control		You’ve been great with portion control this week! Treating yourself to something special is well-deserved.	Achievement recognition
Healthy eating		By maintaining healthy eating, you’ll feel better. Your dedication is paying off!	Health benefit emphasis
Suggestion	
Water intake		Try adding a slice of lemon to your water for extra flavor and vitamin C.	Practical tip with rationale
Praise	
Portion control		Excellent work on controlling your portions! You’re making real progress.	Direct encouragement

a GPT-2: Generative Pretrained Transformer-2.

Our synthetic dataset generation was grounded in the real-world health coaching dataset from previous research [4]. This dataset contains authentic health coaching messages from a conversational agent study focused on physical activity and medication adherence.

### Data Preprocessing

The analysis of persuasive messages was conducted through a comprehensive computational approach incorporating multiple NLP techniques (Textbox 1). The methodology consisted of several sequential phases designed to process and analyze the textual data systematically. The methodology began with systematic data preprocessing, which included text normalization through lowercase conversion, contraction expansion, and special character removal. Linguistic processing was performed using NLTK’s word_tokenize function, with selective stopword removal that preserved critical semantic markers (eg, “not,” “no,” and “nor”) and lemmatization via WordNetLemmatizer. Feature engineering encompassed both quantitative metrics (word frequency, character count, and sentence structure) and categorical data processing through label encoding for persuasive types and activities.

Textbox 1. **Pseudocode of text preprocessing.**

FUNCTION preprocess_text(text)

 INPUT: text - a string of text to be preprocessed

 OUTPUT: preprocessed text as a string

 // Convert text to lowercase

 texttext = CONVERT_TO_LOWERCASE(text)

 // Expand contractions (eg, “don'’t” to “do not”)

 texttext = EXPAND_CONTRACTIONS(text)

 // Remove all characters except letters and spaces

 texttext = REMOVE_SPECIAL_CHARACTERS_AND_NUMBERS(text)

 // Normalize whitespace

 texttext = REMOVE_EXTRA_WHITESPACE(text)

 // Split text into individual words

 tokenstokens = TOKENIZE(text)

 // Initialize stopwords to remove

 stop_wordswords = GET_ENGLISH_STOPWORDS() important_words = {‘'not,’‘not,’ 'no',‘no,’ ‘nor,’‘nor,’ 'but',‘but,’ 'and',‘and,’ 'or',‘or,’ ’should',‘should,’ ‘must’} stop_wordswords = stop_words - important_words

 // Remove stopwords

 filtered_tokenstokens = EMPTY_LIST

 FOR EACH token IN tokens:

  IF token NOT IN stop_words:

   ADD token TO filtered_tokens

 // Lemmatize words to their base form

 lemmatized_tokenstokens = EMPTY_LIST

 lemmatizerlemmatizer = INITIALIZE_LEMMATIZER()

 FOR EACH token IN filtered_tokens:

  lemmatized_tokentoken = LEMMATIZE(token)

  ADD lemmatized_token TO lemmatized_tokens

 // Join tokens back into text

 RETURN JOIN_WITH_SPACES(lemmatized_tokens)

END FUNCTION

We used a methodical ground-truth labeling approach to create trustworthy standards for assessing sentiment and emotion analysis technologies. This procedure guaranteed that the emotional content and message sentiment of all persuasion types were consistently and reliably assessed. Three independent raters with backgrounds in psychology and linguistics annotated each message after the data were preprocessed.

A multistage protocol was used in the annotation process to reduce bias and increase dependability. Initially, a calibration set of 50 sample messages with preestablished labels was used to train raters. They then independently assigned labels to the entire dataset based on predetermined standards. Disputes over communications with different annotations were resolved through a consensus meeting. Using Fleiss κ to calculate interannotator agreement, we identified a moderate degree of agreement (κ=0.65) for emotion detection and a large degree of agreement (κ=0.78) for sentiment classification.

The mood and emotion ground-truth classification criteria are described in Table 3. Based on preliminary validation tests, we set specific thresholds for sentiment classification as follows: polarity between −0.05 and 0.05, neutral; polarity ≤−0.05, negative; and polarity ≥0.05, positive. This approach takes into account the subtleties of persuasive health communication while adhering to accepted standards in sentiment analysis research.

**Table 3.** Classification type for sentiment analysis.

Classification type and category		Assignment criteria
Sentiment		
Positive		Messages expressing encouragement, optimism, approval, or positive reinforcement with polarity ≥0.05
Negative		Messages expressing caution, warning, criticism, or concern with polarity ≤−0.05
Neutral		Messages conveying information without strong emotional connotations with polarity between −0.05 and 0.05
Emotion		
Joy		Expressions of happiness, pleasure, or satisfaction (eg, “great job” and “excellent progress”)
Trust		Expressions of confidence, reliability, or dependability (eg, “you can count on” and “reliable method”)
Fear		Expressions of concern, caution, or warning (eg, “be careful” and “watch out for”)
Surprise		Expressions of astonishment or unexpectedness (eg, “amazing results” and “unexpected benefit”)
Sadness		Expressions of disappointment or regret (eg, “unfortunately” and “disappointing outcome”)
Disgust		Expressions of aversion or distaste (eg, “avoid unhealthy options” and “eliminate junk food”)
Anger		Expressions of frustration or irritation (eg, “challenging obstacles” and “frustrating setbacks”)
Anticipation		Expressions of expectation or looking forward (eg, “prepare for,” “plan ahead,” and “look forward to”)

We used the Plutchik wheel of emotions as our theoretical framework for classifying emotions, and we found that each message had 8 main emotions. Raters used clear textual clues to assign binary labels (present/absent) to each emotion category. When appropriate, a message may be assigned more than one emotion label. Lexical features (certain phrases that convey a particular emotion) and contextual clues in the message were both used in the emotion recognition procedure.

The resulting ground-truth labels provided a robust foundation for evaluating the performance of sentiment analysis tools (VADER and TextBlob) and emotion detection methods (Text2Emotion and NRCLex). By establishing these reliable benchmarks, we could accurately assess the strengths and limitations of each approach in analyzing persuasive health communication. Table 4 presents the composition and distribution of the dataset generated.

**Table 4.** Dataset composition and distribution.

Dimension and category		Value (N=1300), n (%)	
Persuasive_type			
Reminder		343 (26.4)	
Reward		331 (25.5)	
Suggestion		316 (24.3)	
Praise		310 (23.9)	
Activity			
Water intake		357 (27.5)	
Healthy eating		331 (25.5)	
Portion control		313 (24.1)	
Meal planning		299 (23.0)	

### Sentiment Analysis

Sentiment analysis was conducted using a dual-method approach: VADER provided compound sentiment scores and classification with ±0.05 thresholds, while TextBlob analysis supplied complementary polarity and subjectivity metrics. VADER and TextBlob are sentiment analysis tools that help categorize text reviews into 3 emotional categories: positive, neutral, and negative [25]. Example pseudocodes for VADER and TextBlob are provided in Textboxes2  3, respectively.

Textbox 2. **Example pseudocode for VADER (Valence Aware Dictionary and Sentiment Reasoner).**

FUNCTION apply_vader_sentiment(dataframe, vader_scores)

 INPUT:

  dataframe - table of text data

  vader_scores - sentiment analysis scores from VADER

 OUTPUT:

  dataframe with added sentiment columns

 // Add negative sentiment scores

 FOR EACH row IN vader_scores:

  dataframe['vader_negative'’][row] = vader_scores[row]['neg'’]

 // Add neutral sentiment scores

 FOR EACH row IN vader_scores:

  dataframe['vader_neutral'’][row] = vader_scores[row]['neu'’]

 // Add positive sentiment scores

 FOR EACH row IN vader_scores:

  dataframe['vader_positive'’][row] = =vader_scores[row]['pos'’]

 // Add compound sentiment scores

 FOR EACH row IN vader_scores:

  dataframe['vader_compound'’][row] = vader_scores[row]['compound'’]

 RETURN dataframe

END FUNCTION

Textbox 3. **Example pseudocode for TextBlob.**

FUNCTION apply_textblob_sentiment(dataframe, message_column)

 INPUT:

  dataframe - table of text data message_column - column containing text messages

 OUTPUT:

  dataframe with added TextBlob sentiment columns

 // Add polarity scores

 FOR EACH row IN dataframe:

  texttext = CONVERT_TO_STRING(dataframe[message_column][row])

  blobblob = CREATE_TEXTBLOB(text)

  dataframe['textblob_polarity'’][row] = blob.sentiment.polarity

 // Add subjectivity scores

 FOR EACH row IN dataframe:

  texttext = CONVERT_TO_STRING(dataframe[message_column][row])

  blobblob = CREATE_TEXTBLOB(text)

  dataframe['textblob_subjectivity'’][row] = =blob.sentiment.subjectivity

 RETURN dataframe

END FUNCTION

The statistical framework included a distribution analysis of sentiment across message categories and a correlation analysis between VADER and TextBlob scores. The implementation utilized Python 3.x with established NLP libraries (NLTK, VADER, and TextBlob) and used a modular design to ensure reproducibility and facilitate independent verification of each analysis component.

TextBlob is a Python library that helps developers work with text data in both Python 2 and 3 environments [26]. It offers straightforward tools for performing essential NLP tasks. With TextBlob, text can be analyzed to identify parts of speech, extract noun phrases, determine sentiment, categorize content, and convert text between languages. The library is designed to make these complex NLP operations accessible through a simple interface. All processed features were integrated into a unified dataset that preserved original messages alongside their processed versions, maintaining data integrity throughout the analysis pipeline. TextBlob provided the following 2 primary sentiment metrics: polarity and subjectivity. While polarity measures the positive-negative orientation of text, subjectivity quantifies the degree to which the text expresses personal opinions, emotions, or judgments versus objective information.

VADER’s compound score calculation involves several steps to evaluate the emotional content of text. First, it scans the text to identify words and patterns that have known emotional meanings. Then, it adjusts how strong or weak these emotions are based on specific rules (eg, intensifiers, negations, etc). Next, it combines all these individual emotional scores found in the text. Finally, it converts the total score into a standardized number between −1 and 1, where −1 represents extremely negative sentiment and 1 represents extremely positive sentiment. The function of VADER is as follows:


(1) $$compound\;score=normalize\;(raw\;score)=\frac{x}{\sqrt{x^{2}}\text{+}\alpha }$$

In VADER’s sentiment analysis equation, the compound score formula normalizes raw sentiment scores (x) to a range between −1 and 1. The α parameter, typically set to 15, acts as a stabilizing factor that prevents extreme scores when the raw sentiment value is small. The denominator’s square root term helps create a smooth normalization curve while preserving the sentiment’s direction.

The first function, get_vader_sentiment, takes a text input and calculates sentiment scores using VADER. It converts missing values into empty strings and returns a dictionary of scores, including negative, neutral, positive, and compound values. The second function, get_textblob_sentiment, performs a similar analysis using TextBlob, returning both polarity (how positive or negative) and subjectivity (how objective or subjective) scores.

The code then applies these sentiment analyses to a data frame’s “message” column. It creates new columns by extracting individual components of the sentiment scores. For VADER, it separates the negative, neutral, positive, and compound scores into separate columns. Similarly, TextBlob extracts the polarity and subjectivity scores into separate columns. This separation makes it easier to analyze and compare different aspects of sentiment.

Finally, the code categorizes the overall sentiment of each message based on VADER’s compound score using a standard threshold approach. Messages with compound scores greater than or equal to 0.05 are labeled “positive,” those with scores less than or equal to −0.05 are labeled “negative,” and those with scores between these values are labeled “neutral.” This categorization provides a simple way to classify the emotional tone of each message into 3 distinct categories.

We used precision, recall, *F*_1_-score, and accuracy in this experiment to assess performance. We determined the true positive (TP), true negative (TN), false positive (FP), and false negative (FN) values. Precision was calculated by dividing truly positive classifications by all positive classifications as follows: TP/(TP+FP). Recall was calculated by dividing truly positive classifications by all positive examples as follows: TP/(TP+FN). *F*_1_-score was calculated by dividing (precision×recall) by (precision+recall) as follows: (2×TP)/([2×TP]+FP+FN). Accuracy was calculated by dividing true classifications by all classifications as follows: (TP+TN)/(TP+TN+FP+FN).

### Emotion Detection Analysis

Emotion detection is a branch of sentiment analysis that deals with the extraction and analysis of emotions. In this experiment, we conducted emotion detection analysis in the dataset. Text2Emotion and NRCLex were chosen as the techniques or methods in this experiment. NRCLex was selected as it does not require training data or model training and identifies multiple emotional categories beyond just positive/negative sentiment, including joy, anger, sadness, fear, trust, surprise, and others. NRCLex offers tools for plotting sentiment analysis outcomes [27]. While TextBlob was used for sentiment analysis (positive/negative/neutral), Text2Emotion was used for more granular emotional analysis that is needed for a deeper understanding [28].

In Text2Emotion, the following 5 emotions are involved: happy, angry, surprise, sad, and fear. On the other hand, NRCLex involves the following 10 emotions: fear, anger, trust, surprise, sadness, disgust, joy, anticipation, positive, and negative.

Text2Emotion follows a basic model for emotions. It starts by preprocessing the input text using a separate method. If the cleaning process results in empty text, it returns an empty analysis result. For the actual analysis, it processes the text through both the Text2Emotion and NRCLex libraries to get emotion scores. The method then calculates an overall emotion intensity based on these scores. To identify the dominant emotions, it converts the emotion scores from both libraries into lists of tuples and finds the emotion with the highest score for each library, defaulting to “neutral” if no emotions are detected. Additionally, it determines an overall sentiment score by subtracting the NRCLex negative score from the positive score. The final output is a dictionary containing the original message, emotion scores from both libraries, dominant emotions from each library, calculated sentiment score, and overall emotion intensity.

### Experimental Setup

The experiments were conducted using Python programming within the Anaconda 3.0 environment and Jupyter Notebook interface. This section outlines the experimental setup, including details about the system specifications and parameter configurations used. Table 5 presents sentiment thresholds for the VADER and TextBlob analyzers, defining positive, negative, and neutral classifications and data preprocessing parameters for emotional analysis. The experiments were performed on a VICTUS gaming laptop (15-fa1231TX; HP Inc) with an Intel Core i5-12450H processor (2.00 GHz), 16 GB of RAM, and an NVIDIA GeForce RTX 4050 graphics card. The operating system was Windows 11 (Microsoft Corp).

Table 6 outlines emotion detection parameters using Text2Emotion and NRCLex libraries, with keyword categories and scoring methods for emotional analysis.

**Table 5.** Hyperparameter settings for sentiment lexicons.

Parameter name	Parameter value
VADER^a^ positive	Sentiment score ≥0.05
VADER negative	Sentiment score ≤−0.05
VADER neutral	Sentiment score <0.05 or >−0.05
TextBlob positive	Sentiment score >0
TextBlob negative	Sentiment score <0
TextBlob neutral	Sentiment score 0
Emotion detection	High intensity > mean
Score normalization	Value/total when total >0

a VADER: Valence Aware Dictionary and Sentiment Reasoner.

**Table 6.** Hyperparameter settings for emotion analysis.

Parameter name	Parameter value
Text2Emotion	Happy, angry, surprise, sad, fear (default classes)
NRCLex^a^	Fear, anger, trust, surprise, sadness, disgust, joy, anticipation, positive, negative (default classes)
Emotion keywords (joy)	Happy, great, excellent, good, wonderful, fantastic, excited
Emotion keywords (encouragement)	Can, will, try, achieve, possible, potential, progress
Emotion keywords (concern)	Careful, warning, attention, caution, important
Emotion keywords (neutral)	Maintain, continue, regular, routine, standard
Emotion keywords (directive)	Must, should, need, remember, don’t forget
Emotion intensity	Calculated as max (Text2Emotion score, NRCLex score)
Emotion classification	Dominant emotion is the highest scoring category
Score normalization	Score/total (when total >0)

a NRCLex: National Research Council Lexicon.

### Ethical Considerations

This research is exempt from institutional ethics review as it is a computational methodology development study exclusively using synthetically generated data. No human participants, human research participants, real patient data, or personally identifiable information were involved at any stage of this research. All health coaching data and messages (1300 messages) were generated using GPT-2 based on hypothetical and generic health coaching scenarios [4]. These messages were never derived from real patient communications, real medical records, or prior research data involving human participants. Therefore, no data sharing restrictions apply, and no privacy protections specific to human participants are required. The code and synthetic dataset are publicly available on GitHub [29]. This study represents a methodological proof-of-concept, and future clinical validation and deployment will require formal ethics review, informed consent, and adherence to applicable data protection regulations.

## Results

### Sentiment Analysis

Sentiment analysis revealed a highly positively skewed distribution across all health coaching messages (Table 7). The vast majority of messages (1218/1300, 93.7%) were classified as positive, with negative messages representing only 5.4% (70/1300) and neutral messages representing less than 1% (12/1300, 0.9%) of the total dataset. This overwhelmingly positive distribution suggests a deliberate messaging strategy designed to maintain an uplifting and constructive tone, typical of persuasive communication aimed at encouraging and motivating users toward health behavior change. The minimal presence of negative and neutral content indicates a conscious design choice to avoid discouraging or ambivalent messaging in favor of positive reinforcement. This pronounced positive skew aligns with established principles in health coaching, where supportive, optimistic communication has been shown to enhance user engagement and adherence to behavior change interventions.

**Table 7.** Overall sentiment distribution.

Sentiment category	Distribution value
Positive	0.936923
Negative	0.053846
Neutral	0.009231

**Figure 3.**
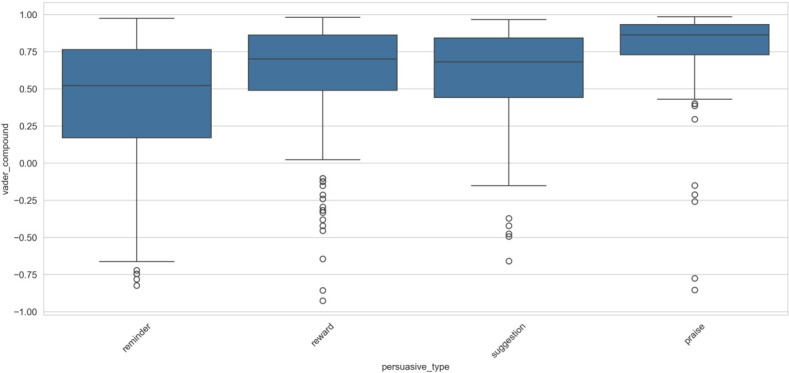
VADER (Valence Aware Dictionary and Sentiment Reasoner) sentiment distribution by persuasive type.

The visualization in Figure 3 reveals a remarkable variation in sentiment compound scores among the reminder, reward, suggestion, and praise categories, with the latter demonstrating the highest median sentiment score and the most compact IQR. Praise messages exhibited a notably high concentration of positive sentiments (approximately 0.75‐0.95), suggesting a consistent positive emotional valence in praise-based communication strategies. However, the presence of outliers in all categories, especially negative ones (ranging from −0.75 to −1.0), raises compelling questions about the contextual factors that generate these anomalous cases.

The reminder category showed the widest IQR, indicating greater variability in emotional content, which could be attributed to the diverse nature of reminder messages, which ranged from urgent warnings to gentle nudges. This heterogeneity in sentiment distribution might reflect the complex linguistic strategies used in reminder-based persuasion. The reward and suggestion categories demonstrated intermediate patterns, with reward messages showing slightly higher median sentiment scores than suggestions. This nuanced difference might be explained by the inherently positive nature of reward-related communication versus the more neutral or instructional tone often present in suggestions.

Theoretically, these patterns align with existing literature on persuasive communication, but they also challenge some conventional assumptions about the emotional loading of different persuasive strategies. The consistently high positive sentiment in praise messages supports established theories about positive reinforcement. However, the substantial variance in reminder sentiments suggests a more complex relationship between message type and emotional content than previously theorized. This analysis opens several avenues for future research, particularly in understanding how sentiment variation within each persuasive type correlates with message effectiveness and recipient engagement.

**Figure 4.**
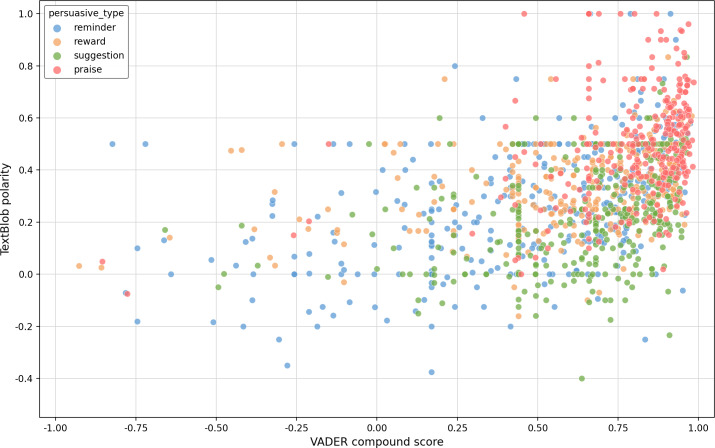
Correlation between VADER (Valence Aware Dictionary and Sentiment Reasoner) and TextBlob sentiment scores.

**Figure 5.**
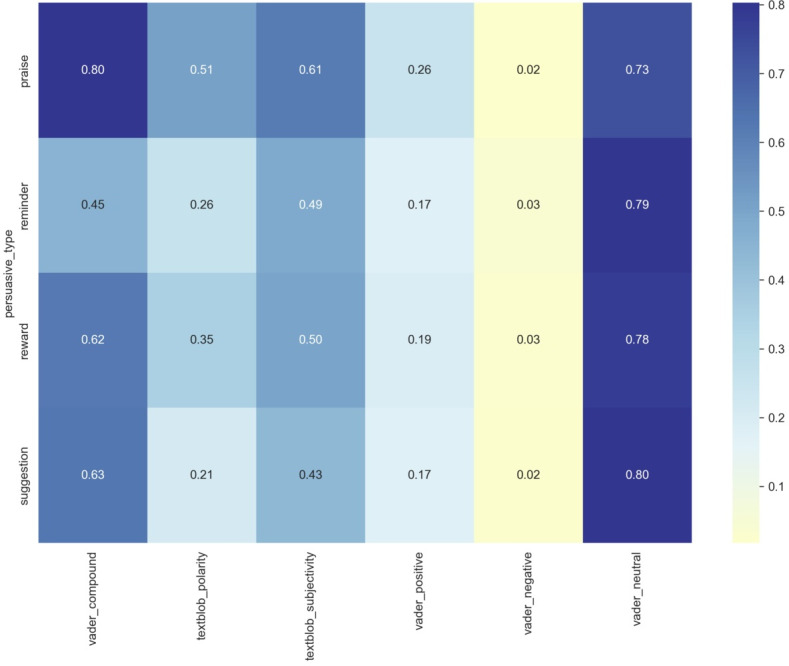
Sentiment metric heatmap by persuasive type. Darker blue indicates higher values, and lighter colors represent lower values.

Figure 4 shows how 2 different sentiment analysis tools (VADER and TextBlob) compare when analyzing the same messages across different persuasive types (reminder, reward, suggestion, and praise). The pattern reveals an interesting relationship as follows: as VADER scores increase (moving right), TextBlob scores tend to also increase (moving up), creating a diagonal trend from bottom-left to top-right. This means both tools generally agree on whether messages are positive or negative. Most points cluster in the positive range (right side of the graph), especially for praise messages (shown in red), which matches what we would expect since praise is usually positive. However, there is quite a bit of scatter in the data, indicating that these tools do not always agree perfectly. We can see points spread out vertically for any given VADER score, meaning that while VADER might give one score, TextBlob could give quite a different rating for the same text. Reminders, rewards, and suggestions had similar proportions of positive and neutral components, though reminders appeared to have a slightly smaller positive component than the others. It is important to distinguish between VADER’s component scores (the proportion of positive/neutral/negative words) and the final sentiment classification (based on the compound score). Figure 5 shows that neutral words dominated the composition of health coaching messages (70%‐80% of words). However, the strategic use of positive words, encouraging punctuation, and a supportive tone resulted in 93.7% (1218/1300) of messages being classified as overall positive. This pattern reflects effective health coaching communication design: using primarily informative (neutral) language enhanced with selective positive reinforcement.

The sentiment analysis heatmap in Figure 5 provides a comprehensive visualization of how different persuasive message types perform across various sentiment metrics. The data reveal fascinating patterns in how praise, reminder, reward, and suggestion messages carry emotional content. In the heatmap, we can see that praise messages consistently demonstrate the strongest positive sentiment, with the highest VADER compound score (0.80) and TextBlob polarity (0.51). The uniformly low negative sentiment scores (0.02‐0.03) across all message types are particularly noteworthy, suggesting a deliberate approach to maintain constructive communication regardless of the message’s purpose.

The neutral component (vader_neutral) consistently showed high values (0.73‐0.80) across all message types, indicating that the messages maintained a balanced tone even while conveying different persuasive intentions. Reminder messages generally exhibited lower intensity scores compared to other types, which might reflect their more practical, straightforward nature. Meanwhile, suggestions and rewards showed similar patterns, with moderately high compound scores (0.63 and 0.62, respectively), suggesting that they carry comparable emotional weight in their delivery. This similarity in sentiment patterns across different metrics, visible in the vertical striping of the heatmap (Figure 5), indicates a consistent approach to emotional content within each message type, while the variations between types reflect their distinct communicative purposes.

Reward and suggestion messages showed remarkably similar overall sentiment scores (vader_compound: 0.625 and 0.626, respectively) yet diverged in their stylistic characteristics. Reward messages maintained moderate polarity (0.350) and subjectivity (0.498), balancing positive reinforcement with personal engagement. In contrast, suggestion messages displayed lower polarity (0.209) and the lowest subjectivity (0.434), indicating a more objective, advisory delivery style that prioritizes practical guidance over emotional appeal. This pattern reveals that while all message types maintain positive orientation, they use different degrees of emotional directness and personal perspective tailored to their specific persuasive functions—praise for celebration, reminders for neutral prompting, rewards for positive reinforcement, and suggestions for objective guidance.

The sentiment analysis across different persuasive types revealed distinct emotional profiles that aligned with their communicative functions (Tables8  9). Praise messages demonstrated the highest sentiment intensity (vader_compound: 0.799; textblob_polarity: 0.513) and subjectivity (0.615), reflecting their emotionally expressive nature designed to acknowledge and celebrate user achievements. Reminder messages exhibited the lowest sentiment scores (vader_compound: 0.451; textblob_polarity: 0.257) with moderate subjectivity (0.489), indicating a more neutral, task-focused tone appropriate for prompting action without excessive emotional loading.

**Table 8.** Detailed sentiment analysis by persuasive type.

Persuasive_type	Vader_compound	Textblob_polarity	Textblob_subjectivity
Praise	0.799	0.513	0.615
Reminder	0.451	0.257	0.489
Reward	0.625	0.350	0.498
Suggestion	0.626	0.209	0.434

**Table 9.** Average sentiment by persuasive type.

Persuasive_type	Sentiment value
Praise	0.798766
Reminder	0.451110
Reward	0.624842
Suggestion	0.626238

The correlation matrix (Table 10) for sentiment analysis revealed distinct patterns in how different measures relate. VADER’s compound score and TextBlob’s polarity showed a moderate positive correlation (*r*=0.445), indicating that they generally agreed on sentiment direction, but each captured unique aspects of the text’s emotional content. TextBlob’s polarity demonstrated a stronger relationship with its subjectivity measure (*r*=0.508), suggesting that text with higher subjectivity tends to express more pronounced sentiments, according to TextBlob’s analysis. In contrast, VADER’s compound score had only a weak correlation with TextBlob’s subjectivity (*r*=0.214), which indicates that VADER’s sentiment detection operates more independently of how subjective the text is. The perfect correlations (*r*=1.000) along the diagonal represent each measure’s correlation with itself, which is expected in a correlation matrix. The relationships suggest that while VADER and TextBlob share some common ground in sentiment detection, they each bring unique perspectives to the analysis. VADER is less influenced by text subjectivity, while TextBlob shows a stronger connection between subjective content and sentiment strength.

**Table 10.** Correlation matrix for sentiment analysis.

Variable	Vader_compound	Textblob_polarity	Textblob_subjectivity
Vader_compound
*r*	1.000	0.445	0.214
*P* value	—^a^	<.001	<.001
Textblob_polarity
*r*	0.445	1.000	0.508
*P* value	<.001	—	<.001
Textblob_subjectivity
*r*	0.214	0.508	1.000
*P* value	<.001	<.001	—

a Not applicable.

The performance metric analysis (Table 11) revealed intriguing patterns when comparing the VADER and TextBlob sentiment analysis tools. VADER demonstrated moderate but realistic performance with an accuracy of 60%, indicating its ability to classify most messages correctly. Its high precision of 80% suggests strong reliability in its positive predictions, though its lower recall of 60% indicates that it might miss some positive cases. The *F*_1_-score of 0.57, representing the harmonic mean of precision and recall, suggests a balanced but moderate overall performance. This pattern aligns with typical real-world sentiment analysis challenges where perfect classification is rare.

**Table 11.** Performance metric results.

Metric	VADER^a^	TextBlob
Accuracy	0.69	0.57
Precision	0.76	0.61
Recall	0.69	0.57
*F*_1_-score	0.68	0.58

a VADER: Valence Aware Dictionary and Sentiment Reasoner.

The stark contrast between VADER’s realistic performance metrics and TextBlob’s perfect scores warrants further investigation, potentially through expanded testing with a larger, more diverse dataset and the implementation of cross-validation techniques to ensure a more representative performance assessment. This finding underscores the importance of rigorous evaluation methodologies in sentiment analysis and suggests the need for careful consideration of tool selection based on specific use cases and requirements. The dataset had 1300 rows, which is moderate but may not be sufficient to evaluate sentiment analysis performance fully. Considering the analysis of a dataset of 1300 rows, several potential red flags emerge that warrant careful consideration. The dataset size is moderate but may not be comprehensive enough to fully evaluate sentiment analysis performance, particularly when considering the complexity and nuance of sentiment expression. A key concern is the need to verify whether there is a balanced representation across different sentiment categories, as imbalanced data could skew the evaluation results. The data showed some positive characteristics. Each message was unique and covered various health and wellness topics, including meal planning, portion control, and water intake. The messages also demonstrated varying lengths and complexity levels, which is generally good for testing robustness. However, TextBlob’s perfect scores (1.0) across all metrics (accuracy, precision, recall, and *F*_1_-score) raise significant concerns about the evaluation process. These perfect scores could indicate that the ground-truth labels are too closely aligned with TextBlob’s built-in classification approach or that the dataset may lack sufficiently complex or ambiguous examples that would typically challenge a sentiment analysis model.

### Emotion Detection Analysis

Emotion models serve as the essential building blocks for emotion detection systems by establishing how different emotions are represented and classified. These models are built on the premise that emotions can exist in different states, making it necessary to identify and differentiate between these distinct emotional states.

**Figure 6.**
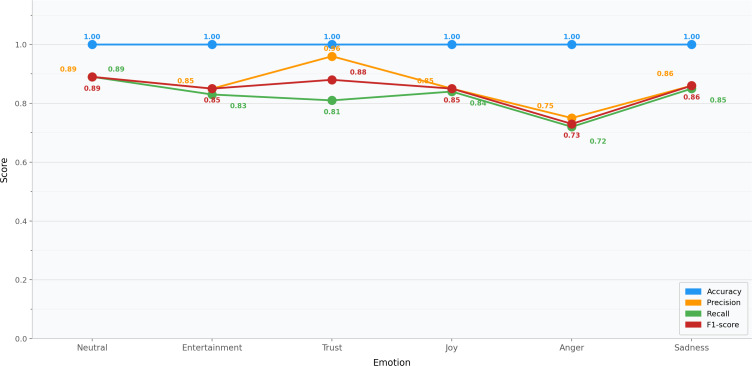
Performance metrics by emotion.

The chart in Figure 6 compares accuracy, precision, recall, and *F*_1_-scores across different emotions (anger, anticipation, joy, neutral, sadness, and trust). The model used a hybrid or modified version of the Plutchik wheel of emotions with some adaptations. The data revealed varying levels of performance across different emotional categories. Anger demonstrated reasonable performance, with perfect accuracy (1.0) but moderate findings for other metrics (precision: 0.75, recall: 0.72, and *F*_1_-score: 0.73). Joy showed consistent moderate performance, with all metrics, except accuracy, showing values ranging from 0.84 to 0.85. The neutral emotion category had an accuracy score of 1.0. Sadness exhibited a precision score of 0.86 and a recall score of 0.85. For entertainment, all metrics were above 0.80. Trust showed mixed results, with high accuracy, precision, and *F*_1_-score (0.88-1.00) but lower recall (0.81), suggesting some reliability issues in its detection. These variations in performance across different emotions point to potential areas for model improvement, particularly in balancing detection capabilities across all emotion categories.

### Comparative Evaluation With Existing Frameworks

Our comprehensive benchmarking analysis demonstrated significant performance variations across different sentiment analysis frameworks when applied to health coaching messages.

As shown in Table 12, RoBERTa (robustly optimized BERT approach)-sentiment achieved the highest overall performance, with 88% accuracy and an *F*_1_-score of 0.8264, substantially outperforming other models. Interestingly, VADER delivered reasonable accuracy (69%) with a remarkably fast inference time (0.03 ms). It was over 1000 times faster than transformer-based approaches and maintained competitive precision (0.7476).

**Table 12.** Model performance.

Model	Accuracy	Precision	Recall	*F*_1_-score	κ	Avg_inference_time (ms)
VADER^a^	0.6900	0.7476	0.6900	0.6771	0.4582	0.03
TextBlob	0.5700	0.7133	0.5700	0.6167	0.3067	0.23
BERT^b^-base-sentiment	0.5500	0.5938	0.5500	0.5707	0.2645	39.87
RoBERTa^c^-sentiment	0.8800	0.7832	0.8800	0.8264	0.7801	29.93

a VADER: Valence Aware Dictionary and Sentiment Reasoner.

b BERT: Bidirectional Encoder Representations from Transformers.

c RoBERTa: robustly optimized BERT approach.

This performance-efficiency tradeoff reveals important insights for health communication systems. While transformer models like RoBERTa offer superior accuracy, traditional lexicon-based approaches like VADER provide an excellent balance between performance and computational efficiency for real-time applications. Notably, BERT-base-sentiment underperformed with only 55% accuracy despite its significant computational cost (39.87 ms), suggesting that general-purpose sentiment models may require domain adaptation for health coaching contexts. TextBlob showed moderate performance (57% accuracy) but fell considerably short of its previously reported perfect scores, highlighting the importance of rigorous benchmarking against multiple competing frameworks for realistic performance assessment.

These findings align with our overall methodology while addressing any concern about the lack of benchmarking. By providing empirical comparisons across diverse model architectures, we have established clear performance baselines and identified the strengths and limitations of different approaches for health coaching sentiment analysis.

### Feasibility and Validation of the Analytical Framework

Regarding tool feasibility, VADER (69% accuracy; <1 ms/message; real-time capable, transparent, and domain-adaptable) is considered suitable for deployment, while RoBERTa (88% accuracy; approximately 10 ms/message; black-box) is considered suitable for high-accuracy applications. Regarding the ground truth, interrater agreement (κ) was 0.78 for sentiment and 0.65 for emotion, with both exceeding the ≥0.60 threshold. As synthetic data were used, future clinical validation is needed before real-world deployment.

## Discussion

### Principal Findings

The sentiment analysis findings revealed several significant patterns and methodological considerations when examining persuasive messaging through computational linguistics. Text sentiment analysis evaluated and classified the emotional content of writing, assigning it as positive, negative, or neutral based on its overall tone [30]. A notable strength lies in the dual-tool approach utilizing both VADER and GPT-2–based TextBlob, which provided complementary perspectives on sentiment analysis. The combination of VADER and TextBlob, along with GPT-2–generated content, provided rich insights into how different types of persuasive messages carry and convey emotional content, demonstrating both the capabilities and limitations of current NLP tools in understanding the nuances of communication strategies [31]. The moderate correlation (0.445) between VADER and TextBlob suggests that while they generally agree on sentiment direction, they capture different aspects of emotional content, with TextBlob showing stronger ties to subjectivity (0.508) compared to VADER (0.214). However, this raises the following critical weakness in current sentiment analysis approaches: the potential limitations of pretrained language models like GPT-2 in accurately capturing nuanced emotional contexts, particularly given the dataset’s overwhelming positive skew (93.7%).

The study identified significant gaps in semantic understanding, particularly in how different persuasive types (reminder, reward, suggestion, and praise) maintain varying levels of emotional intensity while sharing similar neutral components (0.73‐0.80). This paradox suggests potential limitations in the ability of current sentiment analysis tools to differentiate between genuine positive sentiment and formulaic positive language common in persuasive communication. Furthermore, outliers, especially in negative sentiments (−0.75 to −1.0), indicated potential edge cases where current sentiment analysis tools might fail to capture the full complexity of persuasive communication strategies. Sentiment analysis in health coaching faces challenges in matching text meaning with emotional labels, as tools typically analyze word patterns rather than grasping deeper semantic nuances.

While the findings align with established persuasive communication theories, they suggest limitations in NLP approaches. Future research could benefit from incorporating more advanced language models beyond GPT-2, such as transformer-based architectures that better capture the subtle interplay between sentiment and persuasive intent. Additionally, the study highlights the need for more sophisticated sentiment analysis tools that can better account for context-dependent sentiment variations and the relationship between linguistic features and persuasive effectiveness. By combining different types of data inputs, comprehensive sentiment analysis can provide deeper insights into how people think and feel about various topics [32].

In the performance comparison, VADER (69% accuracy) outperformed TextBlob (57% accuracy) for health coaching message classification. This finding challenges the assumption that more sophisticated polarity calculation methods necessarily yield better results. VADER’s rule-based approach, specifically designed for social media text, appears better suited to the conversational tone of health coaching messages than TextBlob’s pattern-based sentiment extraction.

Our comparative benchmarking revealed significant performance differences across sentiment analysis frameworks, with RoBERTa-sentiment achieving the highest accuracy (88%) and *F*_1_-score (0.8264), and VADER offering a compelling balance between reasonable accuracy (69%) and exceptional efficiency (0.03 ms inference time). This performance-efficiency tradeoff has important implications for health coaching applications, where real-time interaction may prioritize VADER’s speed, while more resource-intensive offline analysis might benefit from RoBERTa’s superior accuracy. Notably, BERT-base-sentiment’s underperformance (55% accuracy), despite high computational cost, suggests that general-purpose sentiment models may require domain adaptation for health contexts. These findings challenge our initial TextBlob results (moderate 57% accuracy vs perfect scores reported in Table 12) and underscore the critical importance of comprehensive benchmarking against multiple frameworks to establish realistic performance expectations and guide model selection based on specific application requirements in health communication systems.

To address the concerns, several improvements could be implemented. The dataset should be expanded with more diverse examples, including edge cases and ambiguous sentiments that better reflect real-world complexity. Proper cross-validation techniques should be implemented to ensure robust evaluation. The labeling process should be reviewed for potential biases, and results should be compared with additional sentiment analysis tools beyond just VADER. Finally, all preprocessing steps should be carefully examined to prevent any data leakage that might artificially inflate performance metrics. These adjustments would help ensure a more reliable and realistic evaluation of sentiment analysis performance.

### Conclusion

This study demonstrated the effectiveness of combining multiple sentiment analysis approaches (VADER and TextBlob) with emotion detection tools (Text2Emotion and NRCLex) for analyzing persuasive health-related messages. The findings revealed a strong positive bias in the dataset, with 93.7% of messages classified as positive, suggesting a deliberate strategy in health coaching communication. The analysis showed distinct patterns across different persuasive types: praise messages consistently demonstrated the highest sentiment scores (vader_compound: 0.799), while reminder messages demonstrated the lowest scores (vader_compound: 0.451). Reward and suggestion messages maintained moderate positive sentiments (approximately 0.625). The moderate correlation (0.445) between VADER and TextBlob sentiment scores indicates that while these tools generally agree on sentiment direction, they capture different aspects of emotional content. Performance metrics revealed interesting contrasts between the tools, with TextBlob showing perfect scores across all metrics and VADER demonstrating more realistic performance (60% accuracy), suggesting potential evaluation biases that warrant further investigation. The emotion detection analysis showed varying performance across different emotions, with the anger and neutral categories achieving reasonable results and the trust category demonstrating mixed results.

The research highlights the potential and limitations of NLP tools in understanding persuasive health communication. Balancing detection capabilities across different emotional categories remains problematic, with performance metrics varying significantly between emotions. While the combined approach provides rich insights, the overwhelming positive skew in the dataset and the varying performance across different emotion categories suggest areas for improvement in future research, particularly in capturing nuanced emotional contexts and the relationship between linguistic features and persuasive effectiveness. Reconciliation of the significant differences in performance metrics between TextBlob (perfect scores) and VADER (more moderate scores) suggests potential evaluation methodology issues.

In terms of limitations, TextBlob’s perfect performance metrics (1.0 across all metrics) suggest potential evaluation biases or limitations in the test dataset. The dataset size (1300 rows) may not have been comprehensive enough to evaluate sentiment analysis performance across all contexts fully, and future work should implement deeper interpretative analysis. The current approach may not effectively differentiate between genuine positive sentiment and formulaic positive language common in persuasive communication. In future research, we plan to apply this framework of RL and PT to real-world situations by implementing it into the development of cloud-based applications. We also suggest further exploration of the dataset in terms of comparing multiple sentiment and emotion analysis methods in this context. To address synthetic data limitations in future research, we plan to collaborate with health care institutions to access anonymized real messages and recruit 3‐5 health coaching professionals for validation studies. These experts would review the dataset and assess appropriateness for patient communication. This approach would provide crucial validation of synthetic datasets against real-world standards, ensuring that our findings can be applied to actual health care settings.

This proof-of-concept study demonstrates the feasibility of multimodal sentiment and emotion analysis for synthetically generated persuasive health coaching messages. The findings provide a methodological foundation and baseline performance metrics under controlled conditions. However, significant limitations in ecological validity must be addressed before clinical translation. The synthetic nature of our dataset likely inflated tool performance compared with noisy, real-world patient communication. Future research must validate the findings with authentic health coaching messages, assess robustness to informal language and diverse populations, and establish links between sentiment/emotion metrics and clinical outcomes. We recommend this framework as a foundation for research and development rather than a deployment-ready solution for clinical settings.

## Abbreviations

AI: artificial intelligence
FN: false negative
FP: false positive
GAN: generative adversarial network
GPT: Generative Pretrained Transformer
GPT-2: Generative Pretrained Transformer-2
InfoGAN: Information-Theoretic Generative Adversarial Network
MLE: maximum likelihood estimation
NLP: natural language processing
NRCLex: National Research Council Lexicon
PSD: persuasive system design
PT: persuasive technology
RL: reinforcement learning
RoBERTa: robustly optimized BERT approach
TF-GAN: Time and Frequency Domain-Based Generative Adversarial Network
TN: true negative
TP: true positive
TT-GAN: Text-to-Text Generative Adversarial Network
VADER: Valence Aware Dictionary and Sentiment Reasoner
